# Isolation of Textile Waste Cellulose Nanofibrillated Fibre Reinforced in Polylactic Acid-Chitin Biodegradable Composite for Green Packaging Application

**DOI:** 10.3390/polym13030325

**Published:** 2021-01-20

**Authors:** Samsul Rizal, Funmilayo G. Olaiya, N. I. Saharudin, C. K. Abdullah, Olaiya N. G., M. K. Mohamad Haafiz, Esam Bashir Yahya, F. A. Sabaruddin, Ikramullah, Abdul Khalil H. P. S.

**Affiliations:** 1 Department of Mechanical Engineering, Universitas Syiah Kuala, Banda Aceh 23111, Indonesia; samsul_r@yahoo.com (S.R.); ikramullah@mhs.unsyiah.ac.id (I.); 2 School of Industrial Technology, Universiti Sains Malaysia, Penang 11800, Malaysia; phunmieoseyemi@gmail.com (F.G.O.); ck_abdullah@usm.my (C.K.A.); mhaafiz@usm.my (M.K.M.H.); essam912013@gmail.com (E.B.Y.); atiyah88@gmail.com (F.A.S.); 3 Department of Industrial and Production Engineering, Federal University of Technology, P.M.B.740 Akure, Nigeria

**Keywords:** textile waste, cellulose nanofibre, green materials, biopolymers, environmental

## Abstract

Textile waste cellulose nanofibrillated fibre has been reported with excellent strength reinforcement ability in other biopolymers. In this research cellulose nanofibrilated fibre (CNF) was isolated from the textile waste cotton fabrics with combined supercritical carbon dioxide and high-pressure homogenisation. The isolated CNF was used to enhance the polylactic acid/chitin (PLA/chitin) properties. The properties enhancement effect of the CNF was studied by characterising the PLA/chitin/CNF biocomposite for improved mechanical, thermal, and morphological properties. The tensile properties, impact strength, dynamic mechanical analysis, thermogravimetry analysis, scanning electron microscopy, and the PLA/chitin/CNF biocomposite wettability were studied. The result showed that the tensile strength, elongation, tensile modulus, and impact strength improved significantly with chitin and CNF compared with the neat PLA. Furthermore, the scanning electron microscopy SEM (Scanning Electron Microscopy) morphological images showed uniform distribution and dispersion of the three polymers in each other, which corroborate the improvement in mechanical properties. The biocomposite’s water absorption increased more than the neat PLA, and the contact angle was reduced. The results of the ternary blend compared with PLA/chitin binary blend showed significant enhancement with CNF. This showed that the three polymers’ combination resulted in a better material property than the binary blend.

## 1. Introduction

Textile waste and synthetic polymers are part of the causative agent of pollution in the environment. The use of synthetic polymer for several industrial applications has increased plastic waste pollution [1]. The use of synthetic polymers for packaging is enormous, and this has been reported to take a larger percentage of plastic waste in landfills and ocean pollution. Among the solution proposed is the development of alternative biodegradable materials for industrial applications. This challenge has spurred the ongoing researches on the development of biodegradable composites [2].

Biodegradable polymers, also called biopolymers, are sourced from both plant and animal origin [3]. The biodegradable properties of biopolymers are majorly due to their sources. Biopolymers are also called biodegradable polymers and are isolated from renewable sources. Biopolymers are used as a replacement for synthetic polymers because of their biodegradable properties. Biopolymers are thermally degradable to carbon and biodegradable by microorganisms to carbon dioxide and water [4]. The biodegradable properties of biopolymers remove the environmental pollution concern of synthetic polymers. The rate of degradation of biopolymers is dependent on the microbial action of the soil where it is disposed of [5]. However, previous research on biopolymers’ properties showed that their mechanical properties are low for packaging applications [6,7,8].

Several researches have been done on biopolymers to enhance their mechanical properties [9,10,11]. Biopolymers such as polylactic acid (PLA), poly (butyl acrylate) (PBA), and chitin has been majorly reinforced for improved mechanical properties. Polylactic acid has been at the forefront of biopolymers used in biodegradable packaging [12]. PLA is studied because of its availability, biodegradability, and eco-friendly properties in the environment. There are two primary methods for producing polylactic acid; the chemical method (polymerisation of lactic acid) and the industrial method (fermentation) [12]. Polylactic acid has different forms such as Poly (l-lactic acid) (PLLA), Poly Lactic-co-Glycolic Acid (PLGA), etc., and this is dependent on the stereoisomers and comonomers. Furthermore, there are low and high molecular weights of polylactic acids. PLA has been reported to be brittle and of low mechanical properties like other biopolymers.

Textile Waste cellulose nanofibre has been frequently used to reinforce biopolymer for enhanced strength [3,13]. Cellulose is usually isolated from plant sources, and the demand for it is on the increase. Recently, a review of textile waste’s possible use showed the possibility of cellulose isolation from cotton fabric waste [14,15]. Textile waste as a source of cellulosic materials is a significant breakthrough because it reduces pollution. However, to date, no comprehensive study has been done using textile waste as reinforcement or fillers in biopolymers. Textile waste accounts for the major percentage of municipal waste, which majorly is comprised of disposed of clothing. Globally, tons of fabric waste is generated and this forms part of the land and ocean pollutants [16].

Cellulose nanofibre has been in used as reinforcement in PLA for packaging applications. Several types of research have been done on the combination of PLA and CNF to produce biocomposite film. Previous studies on PLA/CNF biocomposite films [17,18,19]. However, the PLA/CNF blend has poor miscibility (agglomeration) due to the difference in nature. PLA is hydrophobic, while CNF is hydrophilic. The difference in their nature has resulted in poor miscibility and low mechanical properties. However, a material that has good miscibility (with PLA and CNF) can improve the miscibility between them.

Chitin has been blended individually with both PLA and CNF [19,20,21]. Previous studies on PLA/chitin and chitin/CNF showed good miscibility and dispersion of chitin in PLA and CNF. Chitin is generally hydrophobic, and it is normally expected to have good miscibility with PLA. However, due to the hydroxyl functional group’s presence, it also has good miscibility with CNF with a similar functional group. The compatibilizer effect of chitin, due to its good miscibility with PLA and CNF, was used in this study. The properties of PLA/chitin/CNF have not been reported in the literature. The cellulose nanofibrilated fibre used in this study was prepared using combine hydrolysis and high-pressure homogenisation. In this study, a polylactic acid-chitin blend was reinforced with cellulose nanofibrillated fibre for enhanced mechanical properties. The effect of inclusion of chitin on the morphology of the composite was studied.

## 2. Materials and Methodology

### 2.1. Materials

Practical grade 3505D polylactic acid was purchased from Sigma Aldrich Pasir Panjang Rd, Queenstown, Singapore. The polylactic acid has a tensile and yield strength of 62 MPa and 65 MPa respectively. The specific gravity and melt extrusion temperature of the PLA grade is 1.24, and 55–60 °C respectively. The value of PLA molecular flow rate (MFR), g/10 min (210 °C, 2.16 kg) is 14. Chitosan from prawn was deacetylated until 90% and used in this study. The cellulose nanofibrillated fibre used in this study was prepared from textile fabric waste. The percentage composition of polylactic acid and chitin ratio was kept constant at 90% to 10% based on the work by Nasril et al. [21]. The percentage of textile waste cellulose nanofibrillated fibre varied between 1% and 5% by weight.

### 2.2. Textile Waste Cellulose Nanofibrillated Fibre Isolation and Characterisation

Cellulose was isolated from textile waste (100% cotton-based fabrics) with alkaline hydrolysis based on the modified method of Thambiraj [22]. The cotton fabrics were cut into small pieces of 2–3 cm with a milling saw and using mild alkaline hydrolysis (NaOH) was converted into cotton pulp fibre [23]. The fabrics were heated in an alkaline solution of 25 wt% concentration of sodium hydroxide (NaOH) and 0.2 wt% of anthraquinone (AQ) (all percentages based on the weight of the fibre) at 160 °C for 4 h [24]. The cotton pulp fibre was bleached using ozone (gas flow rate of 0.5 L/min at 30 °C for all experiments), a chlorine-free process to avoid the isolated cellulose toxicity [25]. The isolated cellulose was converted to cellulose nanofibrillated fibre using combined supercritical carbon dioxide explosion and high-pressure homogenisation. The bleached cellulose pulp was exposed to supercritical carbon dioxide extraction for 2 h at 500 bars and a temperature of 60 °C to obtain SC-CO_2_ cellulose microfibrillated fibre. After that, a high-pressure homogenisation of 56 MPa and a 44-homogenisation cycle was used to obtain cellulose nanofibrillated fibre (CNF).

The cellulose nanofibrillated fibre obtained was characterised by particle size analysis, FT-IR analysis, and transmission electron microscope (TEM). The FT-IR analysis was used to confirm the isolation of CNF by studying the functional group present. The FT-IR analysis was conducted using FT-IR EFTEM Libra—Carl Zeiss, Selangor, Malaysia. The CNF was mixed with KBr, pressed into the film, and placed in the FT-IR machine for analysis. The nanosize distribution of the particles was studied with a particle size analyzer (Zetasizer Ver. 6.11, Malvern, UK) under dynamic laser light scattering. The cellulose nanofibrillated fibre was dispersed in water at 1 mg to 5 mL and observed under a laser light.

Furthermore, the nano-size of the obtained CNF was further confirmed with TEM. The freeze-dried samples were first dissolved in water, and a drop from the solution was dispersed in acetone on a copper grid. The CNF was observed at 100 nm and 40 kV potential with TEM Perkin-Elmer, PC1600, Winter Street Waltham, MA, USA. The XRD analysis of the isolated cellulose nanofibre powder was done using PANalytical X’Pert PRO X-ray Diffraction (Malvern Panalytical, Techlink, Singapore) at a diffraction angle range of 2θ = 10° to 70°, 1.540598 for K-alpha 1 and K-alpha 2 wavelength, 45 volts, and a tube current of 40 A.

### 2.3. Preparation of Textile Waste Cellulose Nanofibrillated Fibre Reinforced in PLA/Chitin Biocomposite

Polylactic acid, chitosan and cellulose nanofibrillated fibre were extruded to filament form using twin-screw Process 11 extruder, Thermo Scientific (Waltham, MA, USA) at 100/min and temperature profile range of 120 to 180 °C. The extruded filament was pelletised using Thermo Scientific Varicut Pelletizer 11M (Thermo Fisher Scientific, Waltham, MA, USA) and the pellets were hot-pressed using a Carver Press (model 3851-0) (Carver, Wabash, IN, USA) compression moulding machine at 170 °C. The compressed composites were cut to test samples and were stored in a zip lock bag. The percentage by weight between PLA and Chitin was kept constant at 90:10 (Table 1) from previous works [8,11,20,21]. Furthermore, the percentage of cellulose nanofibrillated fibre vary between 0 to 5% by weight of the composite. The percentage of CNF added to the composite was limited to 5% based on previous studies on PLA/CNF [19,26,27].

### 2.4. Characterisation of Textile Waste Cellulose Nanofibrillated Fibre Reinforced in PLA/Chitin Biocomposite

The tensile properties of the neat PLA, PLA/chitin, and the PLA/chitin/CNF were measured with MT1175 (Dia-Stron Instruments, Andover, UK) Instron Universal Testing machine at a force of 50 KN and ASTM 638. The test samples were cut into a dumbbell shape of a standard dimension 165 mm × 19 mm × 3 mm, and the values of five (5) replicates of the samples were documented. The tensile strength, elongation, and tensile modulus were obtained from the test. Furthermore, the fractured surface of the tensile samples was observed to study the miscibility of the polymers.

The impact properties of the neat PLA, PLA/chitin, and the PLA/chitin/CNF biocomposite were tested with Ceast Resil 7181 Impactor (Corporate Consulting, Service and Instruments (CCSI), Akron, OH, USA). The samples were prepared based on D256 standard sizes for impact testing. The value of the impact was obtained in Joule per meter square for five (5) replicates of each sample.

The morphology of the tensile fractured surface of neat PLA, PLA/chitin, and the PLA/chitin/CNF biocomposite was observed with scanning electron microscopy. The scanning electron microscopy (SEM) samples were coated with gold for improved conductivity. The fractured surface images at a magnification of 100 µm were observed with scanning electron microscope EVO MA 10, Carl-ZEISS SMT, Oberkochen, Germany. The XRD was conducted with the powdered form of the neat PLA and PLA/chitin/CNF biocomposite. PANalytical X’Pert PRO X-ray Diffraction (Malvern Panalytical, Techlink, Kaki Bukit Rd, Bedok, Singapore) was used at a diffraction angle range of 2θ = 10° to 50°, 1.540598 for K-alpha 1 and K-alpha 2 wavelength, 45 volts, and a tube current of 40 A.

The FT-IR analysis was conducted using FT-IR EFTEM Libra—Carl Zeiss, Selangor, Malaysia. The CNF was mixed with KBr, pressed into the film, and placed in the FT-IR machine for analysis. The nanosize distribution of the particles was studied with a particle size analyzer (Zetasizer Ver. 6.11, Malvern, UK) under dynamic laser light scattering.

The DMA properties of the neat PLA, PLA/chitin, and PLA/chitin/CNF were conducted at ASTM D4065 standard. The dynamic mechanical analysis of the samples was analysed to evaluate the thermomechanical properties of the material. The test was done with DMA analyser PerkinElmer Dynamic Mechanical Analyzer (DMA 8000) (PerkinElmer Inc., Akron, OH, USA) and the properties of the neat PLA and the PLA/chitin/CNF biocomposites were obtained with changing temperature. The value of storage modulus, loss modulus, and loss factor were obtained with temperature change for each sample.

The thermal properties of neat PLA, PLA/chitin, and the PLA/chitin/CNF biocomposites were studied with thermogravimetry analysis (TGA) and derivative thermogravimetry analysis (DTA). PerkinElmer TG-IR-GCMS Interface Q500, TA Instruments (PerkinElmer Inc., Akron, OH, USA) was used at a temperature range of 40 °C to 800 °C and a 20 °C/min temperature increase. A mass range of 5 mg to 10 mg of the neat PLA and Biocomposites was used as the TGA samples. The rate of degradation (mass decrease) of the biocomposite with temperature was recorded and analysed.

The biocomposite wettability properties were studied with contact angle and water absorption measurements. The samples’ surface contact angle with water was observed with KSV CAM 10 (KSV Instruments Ltd., Espoo, Finland) machine, and the contact angle measurement for five replicates of each sample was obtained. The average contact angle of the neat PLA and the biocomposites were documented. The water absorption test was conducted with ASTM D570 standard for the neat PLA and the biocomposite samples to measure the rate of water intake. The amount of water absorbed by each sample within a 24 h interval was measured using Equation (1)

(1) $$\mathrm{water}\;\mathrm{absorbed}\;\left(\%\right)=\frac{W_{2}-W_{1}}{W_{1}}\times 100$$

where *W*_1_ and *W*_2_ are the initial and final weight of the samples before and after 24 h.

## 3. Results and Discussion

### 3.1. Properties of Textile Waste Cellulose Nanofibrillated Fibre

**Figure 1:**
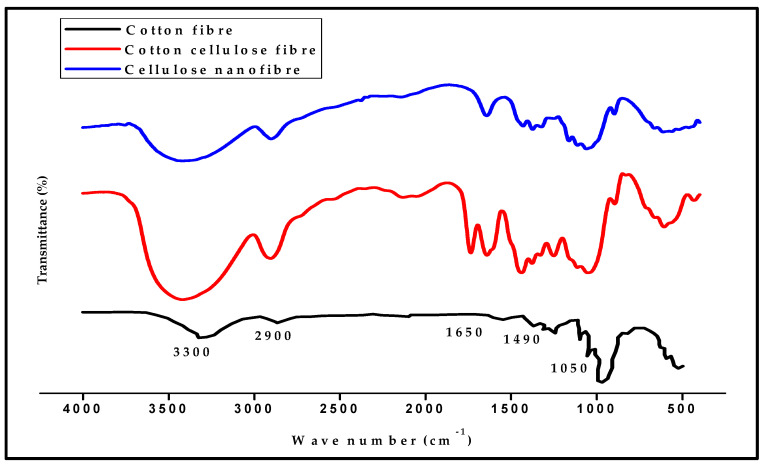
Fourier-transform infrared spectroscopy (FT-IR, Fourier-Transform Infrared Spectroscopy) analysis of Cellulose nanofibre extracted from textile waste fabrics.

Cellulose nanofibre isolation was confirmed with the FTIR functional group analysis and transmission electron microscopy (TEM). Figure 1 showed the result of the FT-IR analysis of the isolated cellulose nanofibrillated fibre from cotton. The FT-IR graph showed the absorption spectra of a typical bond present in cellulose nanofibrillated fibre. The stretched peak between 3200 to 3500 represents the OH bond, the 2500–3000 cm^−1^ band represented the C–C bond, and 1500 to 2000 cm^−1^ the C–H. The peaks at 1050 cm^−1^ is the C–O stretching vibrations. The band between 400 to 800 cm^−1^ is often referred to as the amorphous band of the nanocellulose. These peaks are typical of the chemical bonds present in nanocellulose, as reported in the literature [28]. The analysis showed that using the chemical in the isolation process of CNF in this method does not affect the final product’s chemical structure. The FTIR graph showed similarity to those obtained from other methods as reported. The –OH stretch band is observed to reduce absorbance as the raw fibre approach the complete isolation of CNF. There is no indication of a new band after the isolation process of CNF, which indicates that no new bond was formed with the hydrolysis chemicals to produce cellulose (i.e., digestion and bleaching).

**Figure 2:**
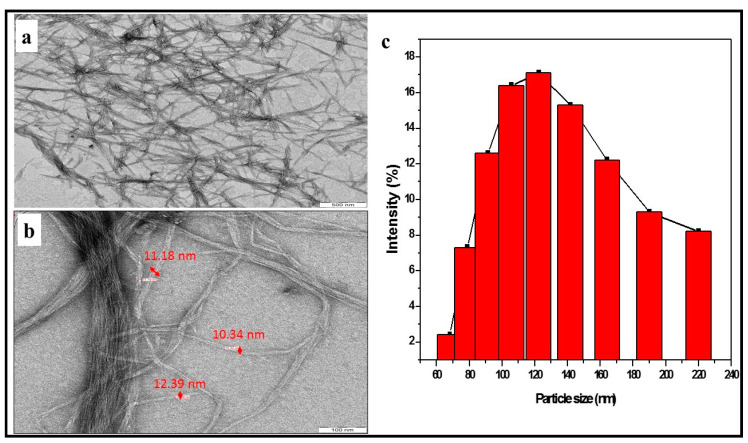
Isolated textile waste cellulose nanofibre transmission electron microscopy image (**a**,**b**) and percentage particle size analysis (**c**).

Furthermore, the transmission electron microscopy and fibre size distribution of the isolated cotton cellulose nanofibre is shown in Figure 2a–c. The TEM images of cellulose nanofibre are presented in Figure 2a. The figure showed fibre diameter in the nanosize range between 10 nm and 30 nm, as measured using the TEM software fibre diameter measurement. Further confirmation of the fibre size diameter was done using Image J software. The nanofibre revealed internetworking with each other, as shown in the figure. The result of the particle size distribution of the isolated cellulose nanofibre is presented in Figure 2c. The particle sizes are measured on a percentage scale, and the intensity (%) of each particle size is plotted. The particle size analysis result showed a size range of 60 nm to 220 nm with a peak between 100 nm and 120 nm. The result showed a higher percentage of nanofibre between 100 nm and 120 nm, and this confirms the isolation of cellulose nanofibrillated fibre from textile waste [23,29,30].

### 3.2. Characterisation of Textile Waste Cellulose nanofibre Reinforced in Polylactic Acid-Chitin Biocomposite

**Figure 3:**
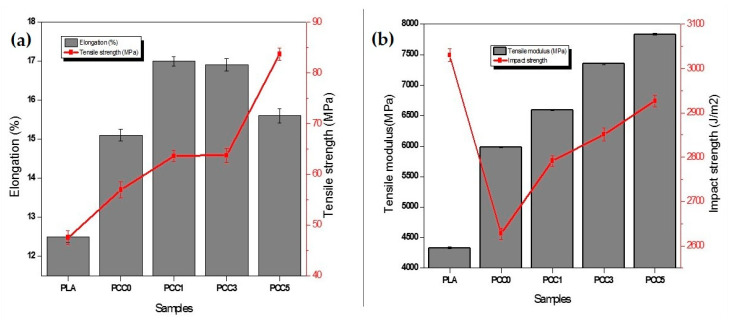
(**a**) Elongation and Tensile strength. (**b**) Tensile modulus and impact strength of neat PLA, PLA/chitin, and PLA/chitin/CNF biocomposite.

The result of the tensile and impact properties of the neat PLA, PLA/chitin, and PLA/chitin/CNF biocomposite is shown in Figure 3a. The tensile strength of the composite is seen to increase with the addition of cellulose nanofibrillated fibre. The highest tensile strength was obtained with PCC5, while the lowest was the neat PLA. The tensile strength values increased significantly from 47.5 MPa to 83.7 MPa. The increase in the tensile strength showed the effect of nanocellulose reinforcement on the tensile properties. A previous report explained that the increase in tensile strength is due to the addition of CNF with a larger surface area. The CNF increased the polymer mix’s bonding, resulting in a well-arranged internal structure of the biocomposite [18].

The elongation (Figure 3a) of the biocomposite showed significant improvement compared to the neat PLA and PLA/chitin. This showed that the addition of CNF also affects the elongation of the biocomposite. However, the elongation is seen to reduce with more CNF though higher than the neat PLA. A similar result is noticed with the extension as they are directly proportional. The elongation trend observed has been reported in the literature with the PLA blend with CNF [17].

A similar trend was observed in the tensile modulus (Figure 3b) of the biocomposite. The tensile modulus increased from 4330.0 MPa to 7836.2 MPa. The tensile modulus value showed a significant effect of the CNF on the PLA/Chitin biocomposite, as observed in the plot. The value of the tensile properties obtained in this result seems to be far higher than those reported in the literature for PLA/chitin [8,21]. This result showed that the addition of CNF further enhanced PLA/chitin’s modulus value compared to PCC1. This makes the ternary blend more suitable for packaging applications compared to PLA/chitin.

The impact test of the neat PLA, PLA/chitin, and the PLA/chitin/CNF biocomposite are shown in Figure 3b. The resilience energy per square metre result of the biocomposite is generally lower than the neat PLA. The addition of chitin resulted in a lower impact strength compared to the neat PLA. The impact energy of the biocomposite range from 2627.25 J/m^2^ for PCC0 to 2927 J/m^2^ for PCC5. The increase in resilience is likely due to the reinforcement effect of the cellulose nanofibre. The resilience of the material is seen to increase with the addition of CNF until PCC5. This shows that the material’s ability to resist impact increased with the addition of CNF, which is needed for packaging applications. The result of the resilience obtained in this study is similar to those reported for PLA/chitin [8,19] and PLA/CNF [19,26]. Previous reports on nanofibre use as reinforcement stated that the fibre use’s aspect ratio affects the resulting composite’s strength. This differentiates the impact strength of composites from each other. Furthermore, the particle size, intermolecular adhesion, and intramolecular cohesion force also affect the polymer blend’s impact strength [19]. Therefore, the increase in this study’s impact strength can be attributed to one or more of these factors.

**Figure 4:**
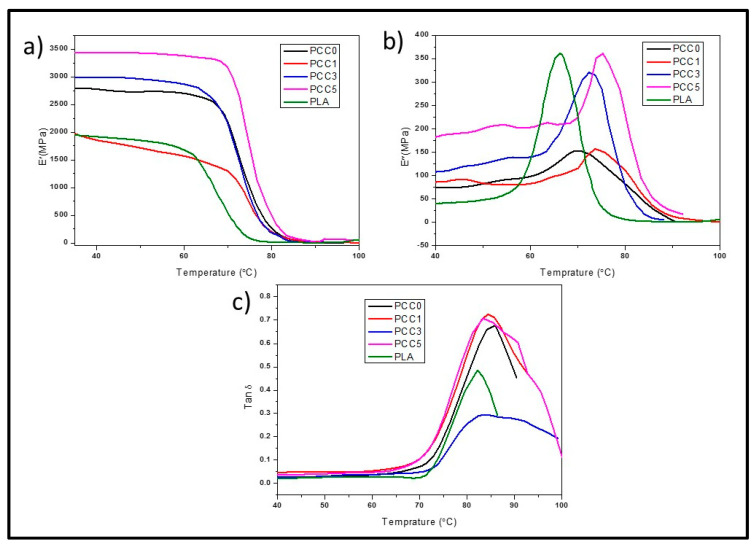
(**a**) Storage modulus (E′), (**b**) loss modulus (E″), (**c**) loss factor (tan δ) of dynamic mechanical analysis for neat PLA, PLA/CNF, and PLA/chitin/CNF biocomposite (PCC1, PCC2, PCC3).

The result of the dynamic mechanical analysis (DMA) of the neat PLA, PLA/chitin, and PLA/chitin/CNF biocomposite is presented in Figure 4. Figure 4a showed the storage modulus result with a change in temperature for each biocomposite and neat PLA. The storage modulus is observed to increase significantly with the addition of CNF compared to neat PLA. A significant increase in the biocomposite storage modulus compared to the neat PLA indicates interfacial interaction between the matrix and the reinforcement. The storage modulus graph showed a distinct transition from the glassy region to the rubbery region [31]. The storage modulus reduction with temperature in all the samples was uniform with a single slope. The single slope in the rubbery region of the storage modulus graph is a characteristic of good miscibility [32]. The highest storage modulus value was obtained with 5% CNF composition, which showed enhancement of the inter or intra surface interaction with the addition of CNF. This means that the high storage modulus indicates less mobility of the polymer chain. More energy is required to stretch, which results in high tensile strength and modulus in the mechanical properties reported in Figure 3. Furthermore, a high storage modulus has a direct effect on the transition temperature of the material. This was observed in Figure 4c as a shift and widening in the tan delta peak temperature. The tan delta peak temperature value of the biocomposite increased with the addition of CNF. The shifted tan delta peak temperature is also termed the glass transition temperature of the material and indicated thermal properties enhancement of the biocomposite with the addition of CNF [8]. The loss modulus (Figure 4b) indicated the damping properties of the biocomposite. The effect of CNF addition on the biocomposite peak temperature is more obvious in the loss modulus peak. The loss modulus peak showed that the neat PLA peak was the lowest, while there was either a shift or widening peak of the biocomposite. The loss modulus peak is often referred to as the stiffness transition temperature, while the tan delta peak temperature is the glass transition temperature [33]. The value of the storage modulus is observed to be higher than the loss modulus, and this indicates that the biocomposite is mainly elastic.

**Figure 5:**
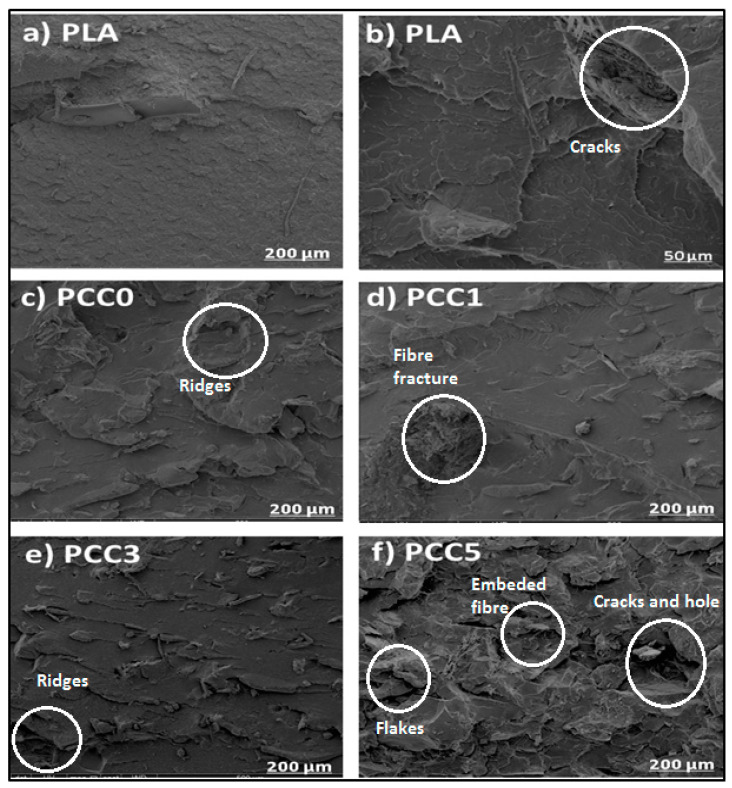
Fractured surface morphology of tensile sample for neat PLA (**a**,**b**), PLA/chitin (**c**), and PLA/chitin/CNF (**d**–**f**) biocomposite.

The result of the tensile fractured surface morphology of the neat PLA, PLA/chitin (PCC0), and the PLA/chitin/CNF (PCC1, PCC3, and PCC5) biocomposite is shown in Figure 5. The morphological images showed good miscibility between biopolymers with no segregation. The images showed increased flakes in the biocomposite morphology with the addition of CNF. Additionally, the fractured surface of the biocomposite becomes rougher compared to the neat PLA. This observation means that the biopolymer blends are thoroughly compacted together with no void. This is probably responsible for the high tensile strength and modulus value observed in the mechanical analysis. The changes in the neat PLA’s morphology when chitin and CNF were added can be significantly seen in Figure 5c to f. The SEM images with the addition of chitin only (PCC0) are characterised with wedges and flakes while the addition of CNF (PCC1, PCC3, PCC5) introduced a fibre network in the SEM images. The network of CNF fibres is shown to increase in the images from PCC1 to PCC5. Previous studies on PLA/CNF biocomposite reported agglomeration of CNF in PLA due to differences in nature [17,18], i.e., PLA is hydrophobic, and CNF is hydrophilic. The difference in the nature of PLA and CNF has resulted in poor mechanical properties, as reported in Reference [18].

However, chitin has been reported to have good miscibility with PLA and CNF. Previous studies on PLA/Chitin and Chitin/CNF have shown good miscibility between these biopolymers [19,21]. In these studies, chitin addition is used as a compatibiliser to enhance the miscibility of the PLA and CNF biocomposite. This compatibiliser effect of chitin resulted in the uniform distribution of CNF in the composite. The miscibility between PLA and chitin is due to the similarity in their hydrophobic nature. In contrast, the miscibility between chitin and CNF is due to the presence of a hydroxyl group in its chemical structure [34]. The morphological images, as observed under scanning electron microscopy showed no void, agglomeration, and segregation. The morphological studies from the SEM corroborated the significant increase in mechanical properties reported in Figure 3. Additionally, the result of the impact strength can be justified from the morphological images. Miscibility and dispersion of the polymer blends can also influence the increase in impact strength [35,36]. The uniform distribution of the CNF in the polymer matrix enhanced the fibre network, which absorbed the impact energy from the impactor [11].

**Figure 6:**
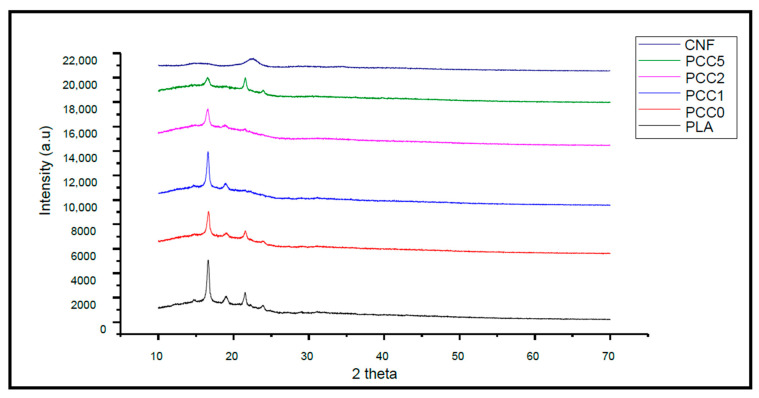
X-ray diffraction analysis of cellulose nanofibre (CNF) extracted from textile waste fabrics, neat PLA, PCC0, PCC1, PCC3, and PCC5.

Figure 6 presents the result of the X-ray diffraction analysis of cellulose nanofibre extracted from textile waste fabrics, neat PLA, PLA/chitin, and PLA/chitin/CNF biocomposite. The crystalline properties of the cellulose nanofibre determine its strength reinforcement ability in the polymer matrix. Distinct peaks were found at two theta equals 15 and 23 degrees, respectively. From previous studies on cellulose nanofibre, similar peaks were obtained [37]. Cellulose nanofibre XRD pattern from the previous study has been reported as highly crystalline with a low amorphous part [38]. Cellulose nanofibre from soft and hardwood has been reported with a 40% to 80% crystalline part [38]. Similar crystallite properties were obtained with the CNF obtained in this study. The crystallinity index was 66.5% using Atiqah et al. [24] method. The crystal peak of the isolated CNF was observed between two theta 10 to 30 degrees, and no significant peak was seen between two theta 30 to 70 degrees, respectively. The XRD analysis confirms cellulose nanofibre’s successful isolation, as established by the FT-IR analysis and previous reports [38].

The X-ray diffraction study of PLA and biocomposites showed a typical characteristic of an amorphous polymeric blend with few crystalline peaks between 15° and 23°. The formation of crystallite between material blends may be due to the PLA’s semi-crystalline properties since the neat PLA showed similar peaks [39,40]. However, the XRD result showed lowered peaks with the addition of CNF, which showed that PLA became less crystalline and this resulted in less brittleness of the PLA [41]. The lowered peaks are probably possible because CNF has both crystalline and amorphous parts. The addition of CNF showed that the XRD peaks were further lowered, mainly due to fibre–matrix interaction. With the addition of chitin only (PCC0), the neat PLA peaks seem not to be significantly affected. This is probably reasonable considering the semi-crystalline of chitin. The brittle nature of PLA is one of its limitations and from a previous study, it has been reported that the addition of chitosan often has no lowered effect on the brittle nature of PLA [21]. However, the result of the XRD showed that biocomposite is more amorphous than crystalline [21].

**Figure 7:**
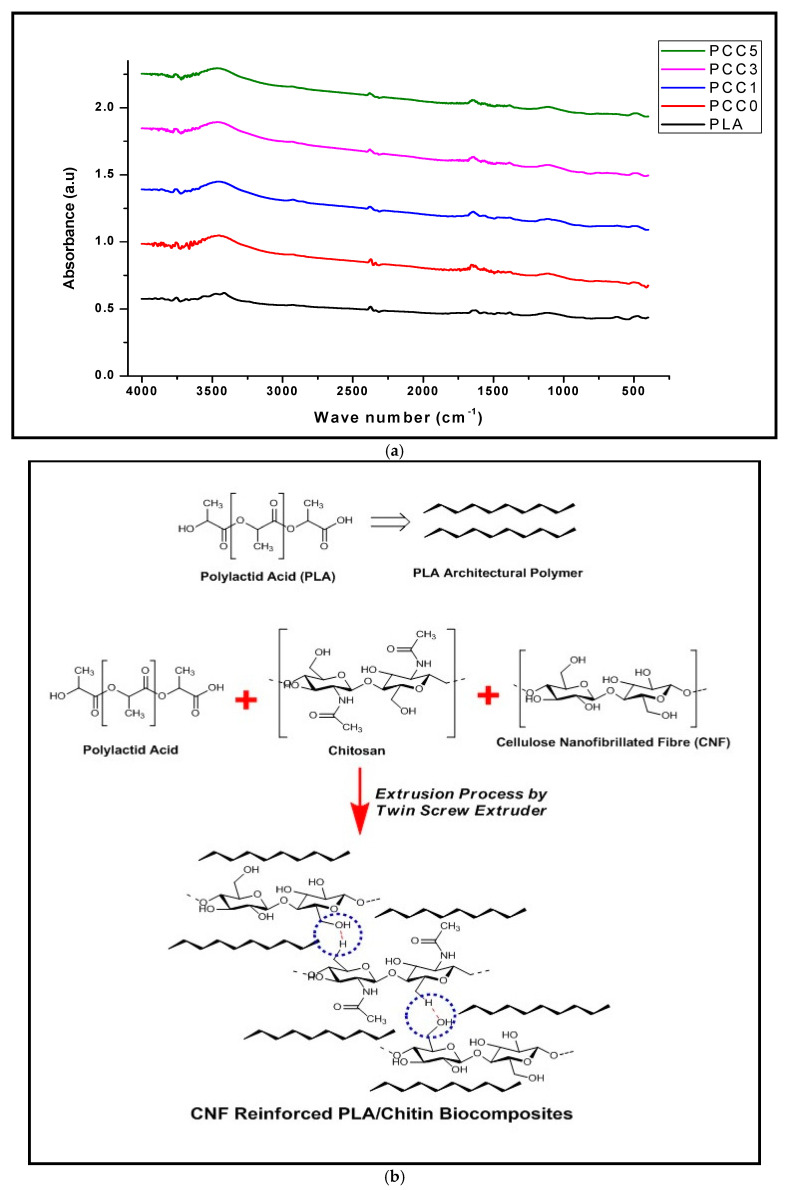
(**a**) FT-IR analysis of neat PLA, PLA/chitin (PCC0), and PLA/chitin/CNF (PCC1, PCC3, and PCC5). (**b**) Chemical reaction and bond formation between the PLA/chitin/CNF biocomposite.

The result of the FT-IR analysis of the neat PLA and the biocomposite (PCC0, PCC1, PCC3, PCC5) is shown in Figure 7a. The FT-IR plot of the neat PLA, PLA/chitin, and biocomposites showed wave numbers between 450 to 4000 cm^−1^. Generally, the FT-IR of the graph showed a major stretch band between 3400–3600 cm^−1^, and small peaks at 2350 cm^−1^, 1650–1700 cm^−1^, and 1150 cm^−1^ wave number. The neat PLA spectra between 3450–3550 cm^−1^, 1700 cm^−1,^ and 1100 cm^−1^ are typical of the lactic terminal, ester group, and vibration of the ester unit, respectively [21,42].

The PLA/chitin (PCC0) spectra band between 3400–3550 cm^−1^ represents the lactic monomer’s combined effect from PLA and the hydroxyl group in chitin. Additionally, the 1650 cm^−1^ in the PCC0 showed C=O and –NH_2_ in PLA and chitin, respectively [21,40]. This is in regards to PLA/chitin/CNF spectra bands. The peak between 3400–3600 cm^−1^ is typical of the presence of –OH hydrogen bonding, 2350 cm^−1^ typical of –C–N, and 1650 cm^−1^ typical of –C=C– bond. The alcohol –OH stretch is typical of hydrogen bonds, resulting from chitin and CNF as both polymers, have the presence of OH in their chemical structure. C–N– bonding in the FT-IR graph is due to chitin’s addition to the blend, while the C=C– is a typically in the presence of the formation of alkene bond between the polymers. The small wavenumber bands below 1000 cm^−1^ showed C–H– bond, common to the three polymeric materials. The presence of –C–N, –OH, and –C=C– is a clear indication of chemical interaction between the three polymeric materials [11,21]. The possibility of bond formation between the three polymeric materials is shown in Figure 7a.

This chemical bonding formation is similar to the one reported in the literature [43]. The FT-IR result showed that enhancement of the biocomposite’s mechanical properties is due to the composite’s physical interaction; the particle sizes of the CNF or the formation of a bond between the three (3) biopolymers. Furthermore, a previous report has shown possible interfacial bonding between PLA and CNF [44,45]. The mechanical properties’ improvement indicates interfacial or intermolecular bonding between PLA, chitin, and CNF.

**Figure 8:**
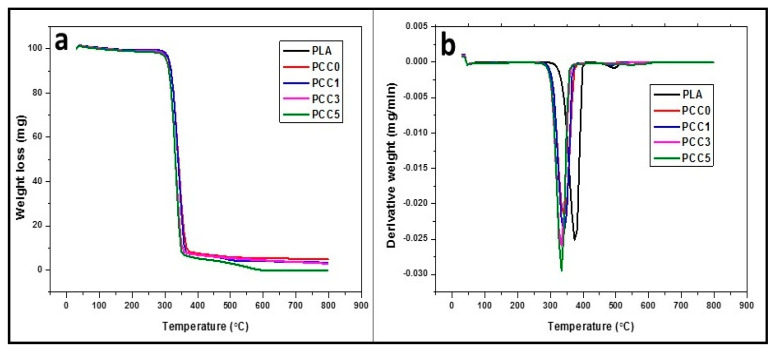
Thermal properties. (**a**) Thermogravimetry analysis (TGA). (**b**) Derivative thermogravimetry analysis (DTA) of neat PLA, PLA/chitin (PCC0), and PLA/chitin/CNF (PCC1, PCC3, and PCC5).

The thermal properties, as analysed with thermogravimetry analysis (TGA) and derivative thermogravimetry analysis (DTA), are shown in Figure 8 for neat PLA, PLA/chitin, and PLA/chitin/CNF biocomposite. The TGA result showed single degradation starting at 276 °C, 280 °C, 284 °C, 290 °C, 298 °C for PCC5, PCC3, PCC1, PCC0, and PLA, respectively.

The TGA onset degradation temperature was observed to reduce with the addition of CNF, while the percentage weight loss of the composite is seen to increase with the addition of CNF. The percentage drop in the neat PLA’s weight, PCC0, PCC1, PCC3, and PCC5 from the thermogravimetry analysis is 91%, 92%, 94%, 96%, and 99%, respectively. The result showed that the percentage decomposition of PLA increases with the addition of CNF, and less residue is found with samples that have a higher percentage of CNF. The TGA graph showed that PCC5 has the highest percentage decomposition and the neat PLA has the lowest. It can be inferred from the result that the addition of CNF to PLA and PLA/chitin improved the biodegradable properties of the biocomposite significantly. The PCC5 has about 8% less residue compared to the neat PLA. As reported in previous literature, chitin’s addition to PLA improved the biodegradability [11,21]. This result showed that the biodegradability of PLA is further improved with the addition of CNF [19]. The degradation properties of the PLA/chitin/CNF biocomposite are a combined effect of chitin and CNF. This result is similar to previous literature on PLA/chitin and PLA/CNF [8,10,17,18].

The DTG result (Figure 8b) also corroborates the finding from the TGA result. The DTG showed that biocomposite thermal stability is lowered with the addition of CNF. This is observed with the peak value of the DTG result. The peak value on the temperature axis of the PCC5 is observed to be less than the neat PLA (Figure 8b). The DTA curve endothermic peak showed values of samples PLA, PCC0, PCC1, PCC3, and PCC5 as 375 °C, 351 °C, 348 °C, 345 °C, and 343 °C, respectively. The peak showed a drop in the biocomposite’s thermal stability with the addition of chitin and CNF [19]. This explains the reason for the increase in the percentage of decomposition. This result showed that the biocomposite becomes less thermally stable at a temperature above the DTG peaks [21]. The peak of the DTG peaks is significant for industrial application, and it determines its thermal limit of usage. The result of the biocomposite’s DTG peak corroborates the observation from the TGA graph, and similar trends have been reported in previous works from the literature [20,21].

**Figure 9:**
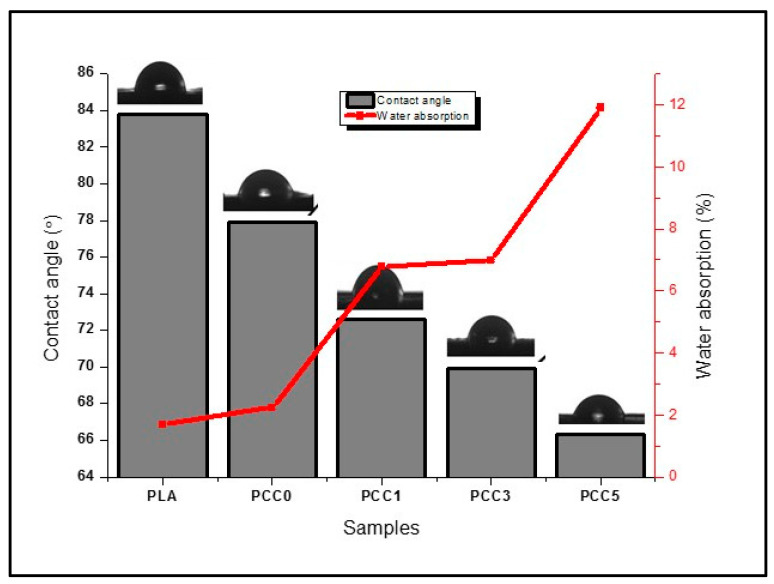
Water absorption and contact angle result of neat PLA, PLA/chitin (PCC0), and PLA/chitin/CNF (PCC1, PCC3, and PCC5).

The result of the water absorption and contact angle analysis of the neat PLA and the PLA/chitin/CNF biocomposites is presented in Figure 9 (line graph). The percentage water absorption of the neat PLA and the biocomposite (PCC0, PCC1, PCC3, and PCC5) increased significantly from 2% (neat PLA) to 12% (PCC5) with the addition of CNF and chitin. The increase in the biocomposite’s water absorption is more significant with the addition of CNF than chitin. The increase in the percentage of the water absorbed can be explained by the nature of CNF being hydrophilic [18]. The addition of CNF increases the ability of neat PLA to form hydrogen bonding with water. This resulted in more water absorption of the biocomposite with the addition of CNF. The effect of the hydrophilic nature of CNF increased the amount of water intake of the neat PLA [11]. Though the water absorption ability of the neat PLA increased, the biocomposite is still highly hydrophobic, based on the value of the water absorbed. The result obtained conforms with previous studies on the PLA biocomposite with chitin and CNF [11,19,21].

Furthermore, the result of the contact angle analysis shown in Figure 9 (histogram) showed that the values are less than 90°. The contact angle values ranged from 83.8° for neat PLA to 66.3° for PCC5, respectively. The contact angle values showed an increase in the biocomposite surface’s wettability with the addition of chitin and CNF. The wettability increase is more significant with the addition of CNF than the addition of chitin, probably because of the hydrophilic nature of CNF [17]. The result of the contact angle also corroborated the result obtained from the water absorption test. Generally, the biocomposite is still hydrophobic from the contact angle [46]. However, there is an increase in wettability. Wettability is an essential property of polymeric materials intended for packaging application. Biocomposite’s hydrophobic nature is needed for the water-repelling properties of the packaged product [12]. The result of the wettability properties of the biocomposite showed that the water repellence of PLA is still retained despite the addition of CNF for mechanical strength enhancement.

## 4. Conclusions

The isolation of CNF was successfully done using combined supercritical carbon dioxide and high-pressure homogenisation. The CNF was used as reinforcement in PLA/chitin/CNF biocomposite, which was successfully prepared with combined melt extrusion and compression moulding techniques. The thermomechanical properties of the enhanced PLA/chitin/CNF biocomposite were analysed. The effect of the CNF on the obtained composite properties was studied and discussed in this study. The result showed improved mechanical, thermal, and wettability properties. The significant improvement in the properties was observed due to the combined effect of the chitin and CNF on the neat PLA. The PLA/chitin/CNF biocomposite result showed its potential use in packaging applications.

## Figures and Tables

**Table 1:** Composition variation.

Sample Name	Polylactic Acid (Wt%)	Chitosan (Wt%)	CNF (Wt%)
PLA	100	0	0
PCC0	90	10	0
PCC1	90	10	1
PCC3	90	10	3
PCC5	90	10	5
